# Multiple immunofluorescence assay identifies upregulation of Active β-catenin in prostate cancer

**DOI:** 10.1186/s13104-019-4100-z

**Published:** 2019-01-30

**Authors:** Pere Puig, Nadina Erill, Marta Terricabras, Isaac Subirana, Judit González-García, Adrià Asensi-Puig, Michael J. Donovan, Lourdes Mengual, M. Teresa Agulló-Ortuño, Mireia Olivan, Antonio Alcaraz, José A. López-Martín, Inés de Torres, José Luis Rodríguez-Peralto, Alfredo Rodríguez-Antolín, Juan Morote, Víctor González-Rumayor

**Affiliations:** 1 R&D Department, Atrys Health, Barcelona, Spain; 2 Icahn School of Medicine at Mount Sinai, MSSM, New York City, USA; 3 Department and Laboratory of Urology, Hospital Clínic, Institut d’Investigacions Biomèdiques August Pi iSunyer (IDIBAPS), Universitat de Barcelona, Barcelona, Spain; 4 Pathology Department, Urology Department and Laboratory of Translational Oncology, Instituto de Investigación Hospital, 12 de Octubre (i + 12), Madrid, Spain; 5 Group of Biomedical Research in Urology, Vall d’Hebron Research Institute (VHIR) and Universitat Autònoma de Barcelona (UAB), Barcelona, Spain

**Keywords:** Active β-catenin, HLA class1, Wnt/β-catenin pathway, Prostate cancer, Systems pathology

## Abstract

**Objectives:**

To apply a systems pathology-based approach to the quantification of nuclear Active β-catenin and human leukocyte antigen class I, and assess the biomarker involvement in a cohort of prostate tumor patients.

**Results:**

The systems pathology approach applied allows a precise quantification of the marker expression in the different cell compartments as well as the determination of the areas that coexpress two markers. Our data shows that the accumulation of β-catenin in the nuclear compartment is significantly decreased in the adjacent normal areas when compared to tumor of the same patients (p < 0.001). In conclusion, the application of this novel multiple immunofluorescence assay demonstrates that the upregulation of Active β-catenin is a relatively common feature of prostate tumor development, and further supports the activation of the Wnt/β-catenin pathway in prostate cancer progression.

**Electronic supplementary material:**

The online version of this article (10.1186/s13104-019-4100-z) contains supplementary material, which is available to authorized users.

## Introduction

The origin of the data is a summary of single observations derived from a more comprehensive study encompassing seven protein biomarkers that were assembled in two multiplex assays and applied to a cohort of prostate cancer patients (n = 505). Quantitative and cellular attributes representing biomarkers from both multiplex assays containing Active β-catenin (ABC), HLA-1 and CK18, among others, were correlated with clinical variables including biochemical recurrence (BCR). One of the objectives of the comprehensive work (manuscript in preparation) was to describe correlations between clinicopathological data and results from the quantification and colocalization of individual marker expressions. When we started analyzing the data we observed that the expression of Active β-catenin was higher in tumor areas than in adjacent normal tissue. In this research note we describe for the first time how a systems biology-based methodology yields a precise quantification of the nuclear Active β-catenin (effector fraction) and thus unequivocally demonstrates the implication of this marker in prostate tumor formation.

Prostate cancer is the most common epithelial malignancy after skin cancer in men from western countries [1]. Systems pathology is a multidisciplinary approach that integrates proteomic, molecular and imaging data with the patients’ clinical history using machine learning [2]. By applying multiplex immunofluorescence and image analysis, we have been able to construct quantitative features for interrogating several biomarkers at the microscopic cellular level. This allows for investigations of co-localization and expression of cellular proteins, specifically during activation of specific signaling pathways. Of paramount importance, the colocalization of a given marker with DAPI can be applied in the quantification of the marker that is present within the nucleus [3].

HLA class I expression on the cell surface of cancer cells is critical for an appropriate anti-tumor immune response by presenting antigenic peptides to cytotoxic T lymphocytes [4]. One of the most frequent escape mechanisms that tumor cells develop to avoid immune system recognition is the loss of HLA-1 expression, which correlates with properties associated with ‘stemness’ resulting in treatment resistance and a poor prognosis [5].

The Wnt/β-catenin signaling pathway is an evolutionarily conserved developmental pathway involved in several physiological processes [6]. Aberrant activation of the Wnt/β-catenin pathway is implicated in many tumor types. β-catenin, a key protein in the Wnt pathway, can trigger the transcription of important regulators of cell cycle progression, cell proliferation and cell stemness upon activation and nuclear translocation [7]. β-catenin expression has been shown to be associated with more malignant, high Gleason score tumors. Both β-catenin and wnt-1 expressions are located in the advancing edge of the tumor [8]. The correlation between higher malignancy related to castrate resistant prostate cancer (CRPC) and β-catenin suggests Wnt/β-catenin signaling pathway upregulation in CRPC [6]. Here we demonstrate the association between nuclear localization of Active β-catenin and prostate tumor progression.

## Main text

### Materials and methods

#### Patient selection and TMA production

Prostate cancer samples were obtained from 505 patients (age range 40–75, median 63 years) with localized and locally advanced disease that underwent a radical prostatectomy (Hospital de la Vall d’Hebron, Hospital Clínic de Barcelona and Hospital 12 de Octubre). The Gleason breakdown of the tumors was as follows: 81 tumors Gleason < 6, 130 tumors Gleason 6, 293 tumors Gleason > 6. Whole tissue sections from each patient were first stained and three tumor cores and a normal core previously selected were included in tissue microarrays (TMAs). Blank 3.5 µM thick consecutive sections of the TMA were used for IF analysis.

#### Multiplex setup

An Immunofluorescent (IF) multiplex reaction was assembled for the study of the following biomarkers: Active β-catenin (ABC), HLA-1 and CK18, described in Table 1, along with the nuclear dye, DAPI (4′,6-diamidino-2-phenylindole, dilactate). CK18 was included to help identify epithelial cells and obtain colocalization values with the other markers. HLA-1 is a member of the HLA system, a cell surface protein family that is responsible for the regulation of the immune system in humans (human leukocyte antigen class I). HLA-1 downregulation is associated with stemness in multiple cancer types [9]. β-catenin Active forms were detected with the mouse monoclonal clone 8E7. Prior to multiplex assembly, individual IF conditions were tested, with selection of the optimal antibody-fluorochrome for each biomarker. The construction of the multiplex required a common heat-induced epitope retrieval (HIER) strategy that incorporated the use of a pH9 solution in a decloaking chamber at 120 °C for 3 min. After HIER, antibodies and Zenons (invitrogen) were added in two addition steps, first HLA-1 combined with ABC, and then CK18. Image acquisition was performed within 48 h. The immunofluorescent multiplex reaction was first analyzed using appropriate cell-line and tissue positive controls. A series of reproducibility experiments were carried out prior to the final conditions applied to the TMA. These setup experiments were used to select the optimal conditions for the image acquisition, as the best filter combination and exposition time for each of the filters. The imaging system included a multispectral camera (Perkin Elmer) attached to a 90i Nikon epifluorescent microscope with an automatized stage controlled by the compatible metamorph^®^ software (Molecular Devices). Multispectral images were acquired at 20× magnification with the Nuance imaging system using the selected filter combination and exposition time, and analyzed with the Nuance^®^ software to unmix and quantify individual biomarker expressions, as well as determining biomarker co-localization in selected regions within each core. The individual expression of each marker is obtained as the result of the quantification of positive pixels for the marker that are included in the tumor area. All expression and coexpression results were obtained as an average of up to three cores from each patient in the case of tumor samples, and directly from a single core of their normal counterpart. Statistical tests were performed after all values were obtained from the multispectral images (see “Statistical tests” subsection).

**Table 1:** Markers included in the multiplex reaction

Antibody	Vendor	Catalog #	Dilution	Isotype	Fluorochrome
HLA class 1	Abcam	Ab52922	1:250	RIgG	R488
Active β-catenin	Upstate	05-665	1:100	MIgG1	M555
CK18	Abcam	Ab82254	1:50	MIgG1	M647

#### Nuclear Active β-catenin assessment

The antibody for Active β-catenin (ABC) is able to detect nuclear, cytoplasmic and membranous ABC. The values for nuclear β-catenin, accounting for the effector fraction of the transcription factor, were deduced from the co-localization between DAPI and ABC in cells that were expressing CK18 (see Additional file 1: Figure S1 for details on subcellular localization of ABC). ABC can be membranous, cytoplasmic and nuclear in cells with activation of the wnt/β-catenin pathway. To avoid any potential overestimation of nuclear ABC due to some background issue related to the cytoplasmic ABC expression a correction value was added. The correction factor, the co-localization value between CK18 and DAPI, is based on the fact that CK18 is only expressed both in membrane and in cytoplasm and should not be found in co-localization with DAPI. Thus, the correction factor should be zero in samples that are not affected by nuclear ABC overestimation.

#### Statistical tests

A Wilcoxon signed-rank test was applied to elucidate differences in the marker expression measures between tumor and adjacent normal areas.

### Results and discussion

#### Marker expression data

**Fig. 1:**
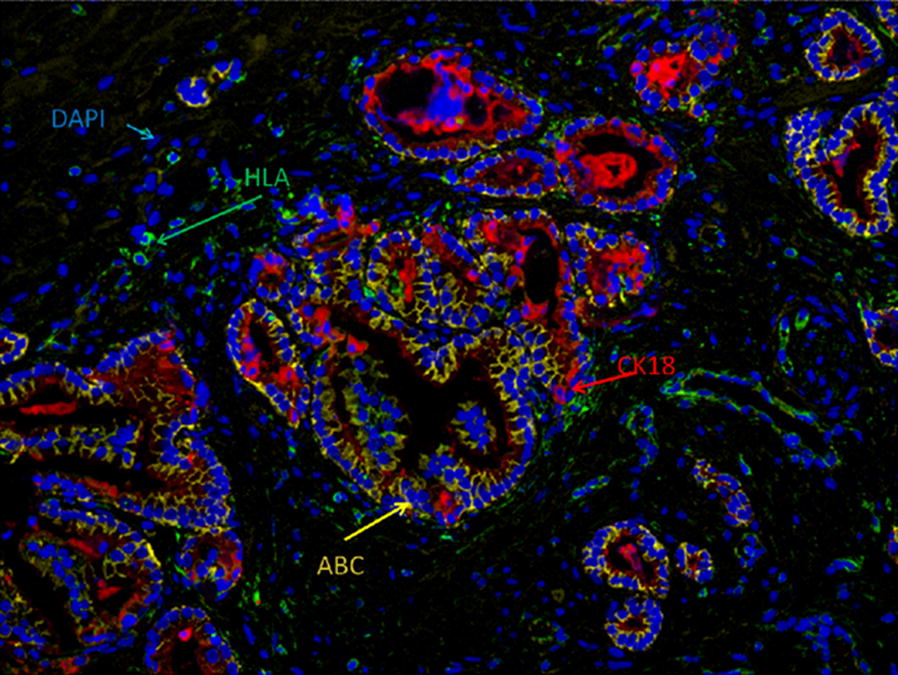
Individual markers with DAPI and multiplex assembling. Multiple fluorochrome labeled antibody hybridization on a prostate sample. Marker and DAPI expressions are shown in different colors; HLA class I in green, CK18 in red, ABC in yellow and DAPI in blue

The immunofluorescent images were acquired and unmixed with the multispectral camera software. After unmixing, the individual marker expression and the coexpression values were obtained from images as depicted in Fig. 1. DAPI, HLA class I, Active β-catenin and CK18 are shown in discrete software assigned colors. DAPI was used as a nuclear counterstain, and its coexpression with a given value applied to the assessment of this marker nuclear fraction. The other markers show a glandular pattern. CK18 is very important in this assay because it is used to both select the glandular area and normalize the expression of the other two markers to CK18 expression. With this normalization, the expression of a marker inferred from the percentage of positive pixels in the fluorescent picture is referred to the amount of glandular compartment. Thus, the glandular normalization contributes to a fair comparison between two images with discordant amounts of tumor or normal glandular areas.

#### HLA Class 1 in tumor formation

HLA class 1 is a critical component of the immune response and a candidate marker to show differences in expression between normal and tumor areas. HLA class 1 downregulation in prostate cancer has been described in previous articles [10, 11]. HLA class 1 expression data was obtained from paired samples using the value for CK18 as a glandular normalization tool, as described in the previous section. Following these criteria 383 out of 505 patients of those included in the cohort were analyzed with a Wilcoxon signed-rank test (Table 2). Our data shows a tendency for HLA class 1 downregulation in prostate tumor samples although the *p* value for the difference between normal and tumor expressions is not as significant (p = 0.025) as expected according to the results described in previous papers [10, 11]. A more extensive cohort may be needed to shed more light on the relation between HLA class 1 downregulation and prostate cancer.

**Table 2:** Wilcoxon signed-rank test for paired samples (n = 383)

Marker	Normal expression Median [Q1; Q3]	Tumor expression Median [Q1; Q3]	p-value
Nuclear Active β-catenin	2.05 [0.00; 7.12]	4.96 [0.46; 10.9]	< 0.001
Total Active β-catenin	0.28 [0.13; 0.58]	0.49 [0.20; 0.89]	< 0.001
HLA class I	0.29 [0.10; 0.70]	0.28 [0.16; 0.50]	0.025

#### Active β-catenin in tumor formation

Nuclear Active β-catenin expression was found associated with prostate tumorigenesis according to the Wilcoxon signed-rank test calculation (p < 0.001). This correlation was obtained comparing the median values of normalized nuclear ABC in the tumor cores with the values of the same normalized ABC fraction in normal cores (n = 383 tumor-normal paired samples). The same level of significance (p < 0.001) was obtained when total ABC expression was assessed and compared in tumor cores vs. normal cores (Table 2).

In the current study, we present nuclear accumulation of Active β-catenin in prostate malignancy development. This finding is in agreement with the published literature [7, 8, 12–14]. β-catenin, a dual effector protein that was first described in the regulation of intracellular adhesion, is also a key nuclear signaling protein in the activation of the Wnt/β-catenin pathway. Downstream targets of β-catenin including c-Myc, Cyclin D1 and CD44, among others, are proliferation agents that are involved in oncogenesis [15]. β-catenin signaling may play important roles in prostate cancer progression [7] and in the acquisition of tumor malignant phenotypes and the capacity for invasion through the induction of AR activity [13].

Interestingly, the correlation between tumor progression and nuclear Active β-catenin is also found with total ABC (nuclear, cytoplasmic and membranous). However, when average expression values are compared, the difference between tumor cores and normal cores is bigger for nuclear Active β-catenin than for total Active β-catenin. Our data further supports a role for the Wnt/β-catenin signaling pathway in prostate cancer formation and as a potential therapeutic target. Furthermore, applications which interrogate biomarkers at the intact tissue-cellular level will continue to advance our understanding of basic tumor biology.

### Conclusions

To summarize, our multiplex-based systems pathology approach is a novel tool for the precise quantification of CK18 epithelial nuclear Active β-catenin through colocalization of DAPI and ABC. The additional evaluation of HLA provides insight into the biology underpinning prostate cancer progression. Thus, the use of the multiplex approach is essential for a more comprehensive analysis of various markers, including DAPI, HLA class I, Active β-catenin and CK18. Our results show a trend for HLA Class I downregulation, and strongly support the implication of Active β-catenin, both nuclear and total, in prostate cancer development.

## Limitations

The study needs an expanded cohort of patients and a more robust assessment of HLA and nuclear ABC to further characterize such discrete cell populations.

## Additional file

**Additional file 1: Figure S1.** Subcellular localization of ABC. Magnified images demonstrating subcellular localization of ABC.

## Abbreviations

ABC: Active β-catenin
BCR: biochemical recurrence
CRPC: castrate resistant prostate cancer
TMA: tissue microarrays
DAPI: 4′,6-diamidino-2-phenylindole, dilactate
IF: immunofluorescent
HIER: heat-induced epitope retrieval
