# Supplementary material for: Multiple immunofluorescence assay identifies upregulation of Active β-catenin in prostate cancer
Source: BMC Res Notes. 2019 Jan 30;12:68. doi: 10.1186/s13104-019-4100-z (PMC6354402; doi:10.1186/s13104-019-4100-z)
Supplement: Supplementary file 1 — Additional file 1: Figure S1. Subcellular localization of ABC. Magnified images demonstrating subcellular localization of ABC. [file 13104_2019_4100_MOESM1_ESM.docx]

Additional figure S1


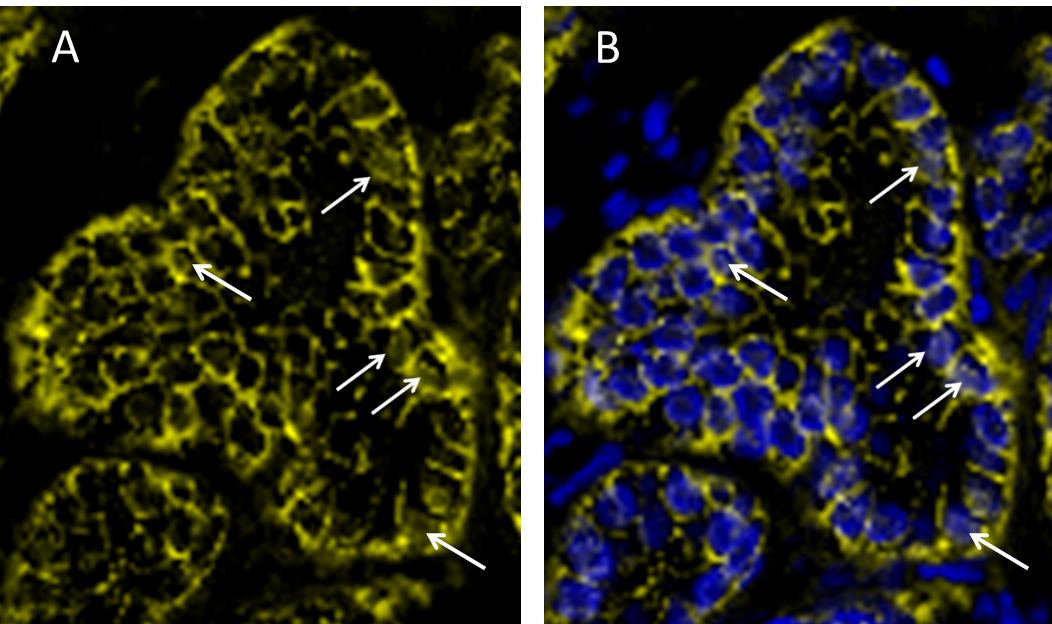


Additional figure S1 Legend

Subcellular localization of ABC. Panel A shows ABC label (yellow) and panel B shows ABC label and DAPI (blue). Nuclear ABC is shown in both panels with white arrows. Nuclear ABC is always detected with a lower intensity than cytoplasmic and membranous ABC.
